# Appointment to the Pennsylvania Hospital

**Published:** 1861-03

**Authors:** 


APPOINTMENT TO THE PENNSYLVANIA HOSPITAL.—Dr. Addinell Hewson has been appointed one of the surgeons to this institution, the post having been rendered vacant by the retirement of Dr. Edward Peace, who had filled it for nearly a quarter of a century.

